# Curvature instability of chiral colloidal membranes on crystallization

**DOI:** 10.1038/s41467-017-01441-3

**Published:** 2017-10-27

**Authors:** Lachit Saikia, Tanmoy Sarkar, Meera Thomas, V. A. Raghunathan, Anirban Sain, Prerna Sharma

**Affiliations:** 1 Department of Physics, Indian Institute of Science, Bangalore, Karnataka 560012 India; 2 Indian Institute of Technology-Bombay, Powai, Mumbai, Maharashtra 400076 India; 3 Raman Research Institute, C.V. Raman Avenue, Sadashivanagar, Bangalore, Karnataka 560080 India

## Abstract

Buckling and wrinkling instabilities are failure modes of elastic sheets that are avoided in the traditional material design. Recently, a new paradigm has appeared where these instabilities are instead being utilized for high-performance applications. Multiple approaches such as heterogeneous gelation, capillary stresses, and confinement have been used to shape thin macroscopic elastic sheets. However, it remains a challenge to shape two-dimensional self-assembled monolayers at colloidal or molecular length scales. Here, we show the existence of a curvature instability that arises during the crystallization of finite-sized monolayer membranes of chiral colloidal rods. While the bulk of the membrane crystallizes, its edge remains fluid like and exhibits chiral ordering. The resulting internal stresses cause the flat membrane to buckle macroscopically and wrinkle locally. Our results demonstrate an alternate pathway based on intrinsic stresses instead of the usual external ones to assemble non-Euclidean sheets at the colloidal length scale.

## Abstract (web summary)

Buckling and wrinkling are instabilities which involve thin elastic sheets and are well-investigated phenomena at the macroscale. Here Saikia et al. investigate curvature instabilities at the colloidal lengthscale in quasi-2D monolayers of rod-like viruses across the fluid-crystal phase transition.

## Introduction

Thin elastic sheets are a common motif found in both biological and synthetic structures^1^. Sufficiently thin sheets undergo curvature instability to buckle out of plane in the presence of compressive stresses^2,3^. The origin of this instability lies in the fact that in-plane stretching energy becomes prohibitively larger than out-of-plane bending energy as the thickness of the sheet is reduced^4,5^. Similar curvature instability also sets in when internal residual stresses appear in the system due to differential (non-uniform) growth or strain^5^. For example, growing leaves and petals^6,7^, torn plastic sheets^8^, and heterogeneous swollen gels^9^ do not remain flat instead spontaneously buckle and develop wrinkles. While shaping of thin sheets through curvature instabilities is a well explored phenomenon at the bulk continuum length scales, it remains an experimental challenge to do the same at the colloidal or molecular length scales^10–13^.

Lipid bilayers and cell membranes are classic examples of molecular thin sheets whose local curvature is typically modulated by external agents such as proteins. Proteins bind to the membranes and cause curvature changes either by imposing their own intrinsic curvature at the site of binding or by distorting the leaflet structure of the membrane through partial insertion^14^. Nanometer length scales and complex heterogeneous composition of cell membranes pose significant barriers in studying cell membrane mechanics. Colloidal monolayer membranes, on the other hand, are much more amenable to detailed investigation due to micron-sized simple controllable building blocks. External boundary constraints like confinement and non-equilibrium phenomena such as drying are some of the strategies employed to modulate their curvature^10,11^. However, internal phase transitions that may drive curvature generation remains an unexplored pathway in the context of molecular and colloidal membranes.

Here, we show the existence of a unique curvature instability that arises during the crystallization of self-assembled colloidal monolayer membranes composed of aligned chiral colloidal rods^15^. Combining complementary microscopy and scattering techniques with suitably chosen assembly conditions, we follow the kinetics of crystallization and associated instabilities in real time by varying the sample temperature. There are two opposing tendencies in the system: inter-rod chiral interaction favoring rod tilting and depletion interaction promoting uniform parallel alignment of rods. The competition between the two results in curvature instabilities during, and on completion of fluid to crystal transition. Such complex crystallization pathways are rarely found in other colloidal systems. We perform discrete Monte–Carlo simulations to validate our understanding of the experimental results. We find that the resultant curvature and surface roughness post crystallization are governed by the size of the membrane and the number of nucleation centers. Furthermore, the crystalline colloidal membranes fracture under external stresses. Unlike conventional dried colloidal films, fractures in these membranes can be annealed by simply tuning the temperature. Our work establishes crystallization as a robust method for making colloidal membranes self-shaping materials with tunable surface roughness and curvature.

## Results

### Phase diagram

**Fig. 1:**
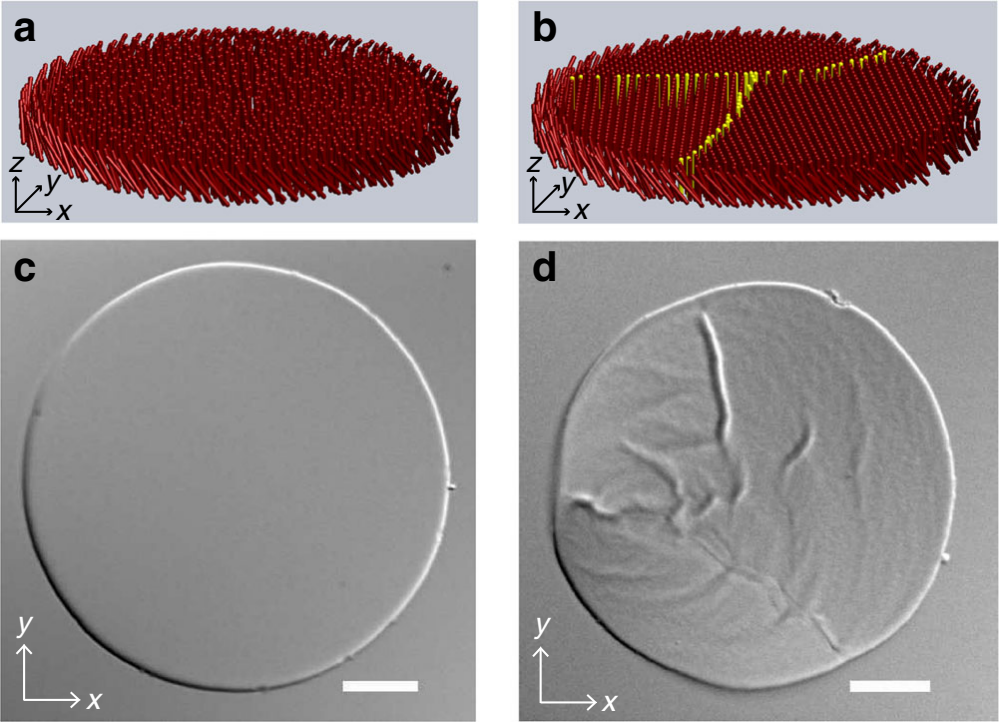
Fluid and crystalline phases of colloidal membranes. **a** Schematic of a fluid colloidal membrane. **b** Schematic of a polycrystalline colloidal membrane assembled with more than one nucleation center. Yellow rods highlight the domain walls. **c** Differential interference contrast (DIC) image of a fluid colloidal membrane at 20 mg ml^−1^ PEG concentration. Scale bar, 10 μm. **d** DIC image of solid colloidal membrane at 24 mg ml^−1^ PEG concentration. Scale bar, 10 μm

Rod-like viruses M13KO7, 1.2 μm in length and 6.6 nm in diameter, spontaneously assemble into monolayers in presence of depletion attraction induced by non-adsorbing polymer Polyethylene Glycol (PEG 35k)^16^. The directional nature of depletion interaction results in all the rods to be aligned along their long axes within the one-rod length thick monolayer membranes (Fig. 1a). At low concentration of PEG (20 mg ml^−1^), the rods are free to diffuse within the membrane. Consequently, the positional order of the rods within these membranes is disordered or fluid-like^16^. Finite line tension causes the fluid membranes to have circular shape whose edges undergo thermal fluctuations at macroscopic length scales (Fig. 1c; Supplementary Movie 1). Theoretical phase diagram of 2D colloidal membranes predicts that rods within the membranes crystallize at high strength of depletion interaction for large enough rod-aspect ratio (Fig. 1b)^15,17^. At higher concentration of PEG (24 mg ml^−1^), we not only find this theoretically predicted solid phase but also discover that the solid membranes are highly roughened and buckled (Fig. 1d; Supplementary Movie 1). Clearly, a hitherto unseen curvature instability arises in conjunction with crystallization of colloidal membranes.

**Fig. 2:**
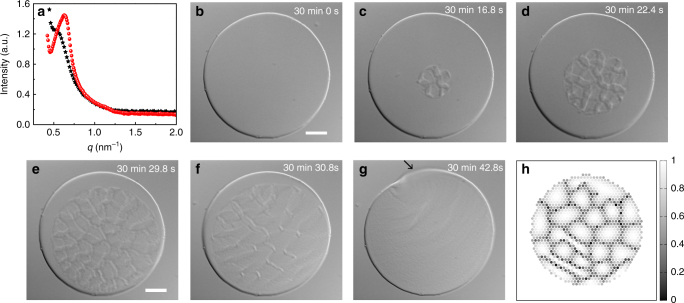
Curvature instability during fluid–crystal transition in colloidal membranes. **a** SAXS intensity as a function of scattering wave vector (*q*) for fluid (black stars) and crystalline (red circles) colloidal membranes at 20 and 25 mg ml^−1^ PEG concentration, respectively. **b**–**d** Time lapse sequence of nucleation and growth of a crystalline domain within a fluid membrane. The growing domain spontaneously develops a network of local ridges (protrusions). Scale bar, 10 μm. **e**–**g** Time-lapse sequence showing annealing of the ridges, shown in **b**–**d**, as the growing domain encounters the edge. The black arrow in the last panel highlights the out of focus edge of the transformed membrane. Scale bar, 10 μm. **h** Grain boundary formation as obtained in the discrete MC simulation using Eq. (1). The plot in grayscale shows the *z*-component of the directors, i.e., $\left( {{\hat {\bf n}}_i} \right)_z \in [0,1]$

We measure the small angle x-ray scattering (SAXS) profiles (Methods) of the fluid and solid colloidal membranes (Fig. 2a) to validate the microscopic structure of the two phases. A broad peak is observed for the fluid phase corresponding to an inter-rod spacing of 11.74 nm. This peak shifts to 10.03 nm in the solid phase, consistent with the expected increase in the density during 2D crystallization^18^. The first zero of the form factor of viruses, calculated by modeling them as cylinders of uniform electron density with a diameter of 6.6 nm, occurs at *q* = 1.16 nm^−1^, which is almost precisely half way between the positions of the (11) and (20) peaks expected from a two-dimensional hexagonal lattice. Therefore, higher order peaks beyond (10) peak become immeasurably small in the SAXS profile of the crystalline phase.

### Mechanism of fluid–crystal transition

We visualize the curvature instability associated with the fluid to solid transition in colloidal membranes by tuning the osmotic pressure of PEG with temperature^19^. At intermediate polymer concentration (22 mg ml^−1^), membranes transform from fluid into solid on cooling to about 15 °C. This transformation is mediated by nucleation and growth (Supplementary Movie 2) as shown by the time-lapse images in Fig. 2b–d. The nucleation time of ~30 min is set by both the degree of supercooling and the overall polymer concentration. The faceted nature of boundary of the growing domain provides further evidence that the solid phase has positional order associated with it. A network of local ridges (protrusions) appear within the growing solid domain as it relieves the excess strain energy associated with dislocations, disclinations, and tilt distortions through out-of-plane buckling (Fig. 2d). As part of the growing domain makes contact with the membrane edge, the local ridges begin to anneal with the rest of the domain continuing to engulf the remaining fluid membrane (Fig. 2e–g). When the membrane transforms into the solid phase in its entirety, it buckles macroscopically as is evident from the partially out of focus edge (highlighted by an arrow in Fig. 2g). The transformation is reversible as the solid membrane melts into the fluid phase when temperature is increased back to room temperature (Supplementary Movie 2). The size of the solid domain increases linearly with time implying interface controlled growth (Supplementary Fig. 1)^20^. The growth rate of the size of the domain was found to be 1.12 ± 0.01 μm s^−1^ when membranes were supercooled to 15 °C. The final morphology of the solid membrane obtained in this time sequence is more uniform and smoother than the one shown in Fig. 1d because of differences in the kinetic pathways of transformation that are discussed below.

### Numerical simulations

The mechanism of local ridge formation shown in Fig. 2b–d can be understood by considering the microscopic chirality of the constituent rods. A single nucleation center in Fig. 2b implies that the grains surrounded by the local ridges are likely to have very small crystalline lattice orientation mismatch, if any. We, therefore, analyze the orientational stability of a 2D hexagonal lattice of chiral rods with their long axes initially oriented along ${\hat{\bf z}}$ using Monte–Carlo simulation of a discrete model for chiral nematics^21,22^. Consider nematic directors ${\hat{\bf n}}_i$ (long axes of the rods) arranged on a 2D hexagonal lattice, where *i* denotes the lattice site. We simulate a finite system, consisting of 2314 rods, and having a circular boundary where torque free boundary condition is imposed^23,24^ (Supplementary Note 1). The energy of the system is given by,

1 $${H = - \epsilon _{\mathrm{N}}\mathop {\sum}\limits_{nn} \left( {{\hat {\bf n}}_i.{\hat{\bf n}}_j} \right)^2  + \epsilon _{\mathrm{C}}\mathop {\sum}\limits_{nn} \left\{ {\left[ {{\hat{\bf r}}_{ij}.\left( {{\hat{\bf n}}_i \times {\hat{\bf n}}_j} \right)} \right]\left( {{\hat{\bf n}}_i.{\hat{\bf n}}_j} \right) - q} \right\}^2 - \epsilon _{\mathrm{D}}\mathop {\sum}\limits_i \left( {{\hat{\bf n}}_i.{\hat{\bf z}}} \right)^2,}$$

where ${\hat{\bf r}}_{ij}$ is the unit vector from the *i*-th to *j*-th director. The sums in the first two terms are over all nearest neighbor (*nn*) pairs (*i*, *j*) and all the terms have ${\hat{\bf n}} \to - {\hat{\bf n}}$ symmetry. The first term promotes nematic ordering as in the Lebwohl–Lasher model^25^ and the third term represents depletion interaction which aligns the rods along the membrane normal (along ${\hat{\bf z}}$). The second term embodies the simplest chiral interaction term $\left[ {{\hat{\bf r}}_{ij}.\left( {{\hat{\bf n}}_i \times {\hat{\bf n}}_j} \right)} \right]\left( {{\hat{\bf n}}_i.{\hat{\bf n}}_j} \right)$ ^26^. We generalized this term to include a preferred chirality *q*, as in continuum Frank free-energy to account for the chiral nature of the viruses^24,27,28^. The nematic and the depletion interaction together promote uniform vertical ordering of the rods along ${\hat{\bf z}}$, while the chiral term opposes it (Supplementary Note 1). Due to this competition, a uniformly oriented state (along the membrane normal) is unstable and spontaneously gives rise to grain boundaries, where the rods have higher inclination angle and higher energy compared to the grain interior (Fig. 2h; Supplementary Figs. 2–5). Experimentally, the grain boundaries locally buckle out of plane and the rods within them gather tilt by following the local layer normal, an effect which our simplistic 2D model cannot capture. The size and the chiral orientation of the grains are controlled by the preferred chirality *q* (Supplementary Figs. 2, 3).

### Dynamics of rods within the colloidal membranes

**Fig. 3:**
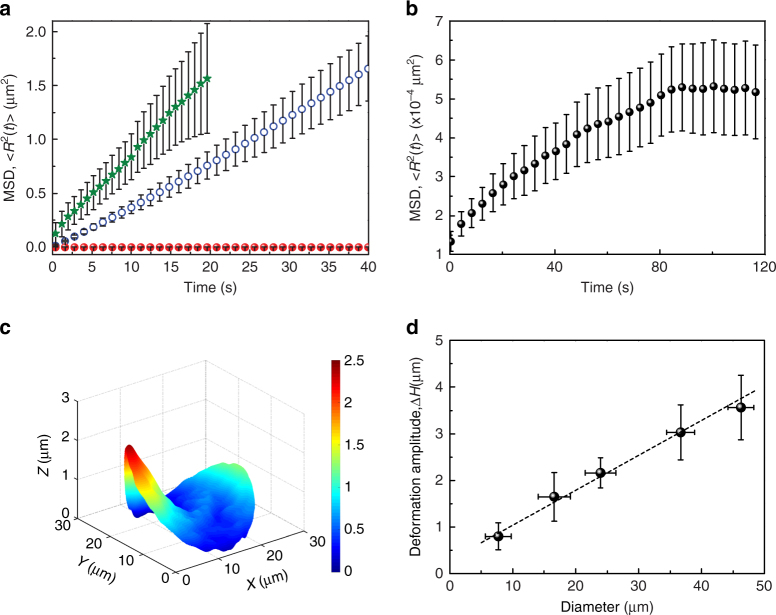
Dynamics and macroscopic structure of crystalline colloidal membranes. **a** Mean squared displacement (MSD) as a function of time for rods within the bulk of a crystalline membrane (red closed circles), bulk of a fluid membrane (blue open circles), and rods at the edge of the crystalline membrane (green stars). **b** Zoomed-in MSD of the rods within the bulk of the crystalline membrane. MSD of rods in the bulk of the crystalline membranes, bulk of the fluid membranes, and edge of the crystalline membranes was obtained by analyzing the individual trajectories of 186, 102, and 50 tracer rods, respectively, present across multiple membranes and multiple sample chambers. The average of all these data sets is plotted in symbols with the standard deviation as the error bar. **c** 3D rendered confocal image of a crystalline colloidal membrane formed out of a single nucleation center. Sidebar shows the color code for *z*-axis values. **d** Deformation amplitude, Δ*H* = *H*_max_ − *H*_min_ as a function of diameter of the colloidal membrane. The raw data corresponding to different membrane diameters were binned to compute a mean deformation corresponding to a mean membrane diameter. The standard deviation of a particular bin has been used as the error bar for that bin

The structure and dynamics of edge of the membrane is responsible for annealing of the local ridges during the phase transformation. We track the motion of single individual rods in colloidal membranes where 1 out of 30,000 rods are fluorescently labeled (bright spots in Supplementary Movie 3, Methods) to follow the dynamics of fluid and crystalline phases. The mean squared displacement (MSD) of rods in the fluid phase is a linear function of time with diffusion coefficient of 0.98 × 10^−2^ μm^2^ s^−1^ (Fig. 3a; Supplementary Movie 3). On the other hand, diffusion coefficient of rods in the bulk of solid membranes is four orders of magnitude lower with a value of 1.3 × 10^−6^ μm^2^ s^−1^, as is expected of in a crystalline phase (Supplementary Movie 3). At long time scales, MSD for these rods saturates to 5.3 × 10^−4^ μm^2^, which is consistent with tilt fluctuations of rods within the membranes (Fig. 3b). While dynamics of rods in the bulk of the membranes is as expected, the rods at the edges of the membranes behave differently. Rods at the edge and in a thin region near the edge are tilted with respect to the layer normal in fluid membranes (Fig. 1a)^29^. The resultant twist penetration depth is set by the microscopic chirality of the constituent rods. This edge-bound cholesteric does not turn into crystalline phase during the transformation due to large energetic barrier associated with untwisting of the rods. It shows complex dynamics, where rods continue to diffuse along the edge and maintain a dynamic equilibrium between attachment and detachment to the bulk solid phase (Supplementary Movie 3 highlighting the edge-bound rods in the circled area). We determine the MSD for these edge bound rods during the time periods, where they remain mobile and find it to be a linear function of time with diffusion coefficient 1.86 × 10^−2^ μm^2^ s^−1^ (Fig. 3a). The edge-bound rods in solid membranes continue to diffuse over days (Supplementary Fig. 6). We can therefore infer that a thin liquid layer encloses the bulk solid membrane.

The chiral interaction energy is lowest for the rods at the edge of the membrane compared to the rods at the local ridges, where chiral interactions are only partially satisfied (Supplementary Fig. 5c). Therefore, the grain within the growing domain that is in contact with edge has lower energy density than all other grains and consequently grows at the expense of others (Fig. 2e; Supplementary Fig. 7)^30^. This causes annealing of almost all the local macroscopic ridges that were present before the membrane edge was encountered by the growing solid domain. This is further supported by the fact that when there are more than one nucleation center in the fluid membranes, all the local ridges can not be annealed completely due to significant mismatch in the crystalline lattice orientations of the growing domains which pose large energetic barriers (Supplementary Movies 5–7).

### Continuum structure and mechanics of crystalline membranes

We measure the extent of buckling, that occurs post annealing of local ridges, by 3D imaging of the solid membranes formed through nucleation and growth from a single nucleation center (Fig. 3c, Methods). Figure 3d shows deformation amplitude, given by difference of maximum and minimum height across the solid membrane, i.e., Δ*H* = *H*_max_ − *H*_min_, as a function of size of the membrane (Supplementary Movie 8). Δ*H* monotonically increases with membrane size (Fig. 3d) as is expected from elastic theory of thin plates^4^.

**Fig. 4:**
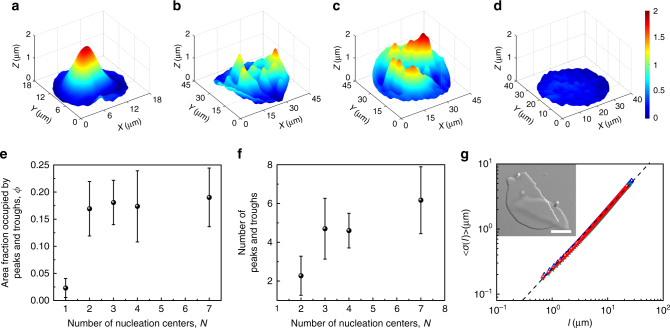
Wrinkling and crack formation in crystalline colloidal membranes. 3D rendered confocal images of crystalline colloidal membranes formed out of chiral fluid membranes containing **a** two, **b** three, **c** four nucleation centers. **d** 3D rendered confocal image of crystalline colloidal membrane formed out of nearly achiral fluid colloidal membrane containing seven or more nucleation centers. All panels have the same color code for *z*-axis values as shown in the sidebar of **d**. **e** *ϕ*, area fraction of crystalline membranes covered by peaks and valleys, as a function of number of nucleation centers. The error bars correspond to the standard deviation of *ϕ*. **f** The number of peaks and troughs within crystalline membranes as a function of number of nucleation centers for membranes formed out of more than one nucleation center. The error bars correspond to the standard deviation of the number of peaks and troughs. **g** Standard deviation of cracked edge profile $\left\langle {\sigma \left( l \right)} \right\rangle$ as a function of box length *l* for three different crystalline colloidal membranes. Dashed line is a fit to the data with a slope of 0.84. Inset shows DIC image of a cracked colloidal membrane. Scale bar, 10 μm

Solid membranes formed through nucleation and growth at more than one site in the membrane show local wrinkling in addition to global buckling. Figure 4a–c show confocal images of the solid membranes that have been formed from varying number of nucleation centers, *N* (Supplementary Movies 4–7). Supplementary Movie 5 shows *N* = 2 case, where solid domains grow from two different nucleation centers simultaneously. A macroscopic ridge (domain wall) gets trapped near the center of the membrane at the end of the crystallization. Membranes formed through *N* > 2 have multiple domain walls within them, which results in local minima and maxima in the height across the entire area of the membranes. Figure 4e shows the fraction of membrane area covered by local peaks and troughs, *ϕ*, as a function of *N* to characterize the change in surface morphology of the membranes (Methods). *ϕ* is lowest for *N* = 1, consistent with the fact that surface topography of such membranes remains locally smooth in absence of any ridges and are buckled at the continuum length scales (Fig. 3c). *ϕ* increases abruptly at *N* = 2 and remains constant for *N* > 2. Though the variation of *ϕ* establishes that membranes formed out of *N* > 2 are all locally rough, the topography of such membranes can be further distinguished by studying the variation of the number of local peaks and troughs with *N* (Fig. 4f). The number of local peaks and troughs increase with increase in *N*.

We study fluid–solid transition in membranes with edge-on configuration (membranes whose layer normal is parallel to the horizontal/coverslip) to determine the effect of steric interactions with the substrate (coverslip) on the curvature instability, if any (Methods). We find that these solid membranes wrinkle in a manner similar to the data reported in Fig. 4 for the face-on membranes (Supplementary Fig. 8).

Solid membranes are mechanically brittle. Gentle external tapping/shaking of the sample chamber is enough to cause fracture in these membranes (inset of Fig. 4g). Fractured membrane edges are jagged and remain so over days (the samples were observed upto 10 days). We characterized their roughness by computing the roughness exponent of the boundary profile of the edge, *Y*(*X*), using root mean square correlation method (Methods). The standard deviation of the boundary profile computed over a window size *l* and averaged over multiple windows, $\left\langle {\sigma \left( l \right)} \right\rangle$ scales as ~*l* ^*κ*^ with roughness exponent *κ* ≅ 0.84 (Fig. 4g), implying that these roughened edges are self affine in a manner similar to those found in rocks, glasses, and metals^31,32^. Interestingly, fractured colloidal membranes are one of the few model systems in which the roughened colloidal interface is still surrounded by the solvent unlike the fractures in dried colloidal films. This enables us to anneal the roughened interface by heating the sample and initiating fluidization (Supplementary Movie 9).

### Role of chirality in the curvature instability

So far this study has used rod-like viruses which are chiral (left handed) in nature. We utilize miscible mixtures of left handed and right hand viruses to address the role played by chirality in the curvature instabilities discussed here^33^. Membranes assembled from miscible mixtures of left handed and right handed viruses are significantly weaker in chirality compared to those assembled from purely left handed ones (Methods)^34^. We find that these membranes remain completely flat, within the diffraction-limited axial resolution, during and on completion of the phase transformation into the crystalline phase (Fig. 4d). Each growing domain remains flat (Supplementary Movie 10) unlike the time lapse sequence shown in Fig. 2b–d (Supplementary Movie 2) wherein local ridges had spontaneously appeared during the growth. The domains walls separating the domains also remain unbuckled. This results in the crystallized weakly chiral membranes to have negligible curvature both locally and globally (Fig. 4d). We, therefore, establish both experimentally and numerically that the chiral nature of the constituent rods is essential for the curvature instabilities to set in.

## Discussion

Crystallization is a fundamental phenomenon that is of both scientific and technological interest^20^. Our experiments on fluid–crystal transition in chiral colloidal membranes have shown that restraining crystallization to two dimensions with shape anisotropic chiral building blocks lead to curvature instabilities. M13 viruses used in these experiments are an ideal model system of hard rods, as demonstrated by a number of theoretical, experimental, and simulation studies^35^. Therefore, our results hold relevance for colloidal or nanorods interacting through excluded volume interactions. The assembly principles presented here are general enough for the assembly of nanorod arrays that have a wide range of potential applications such as solar cells, light emitting diodes, etc. Therefore, understanding the principles that govern the curvature of such arrays is essential for optimizing device applications. Our work also has indirect relevance to cell membrane remodeling, which is usually driven by specialized proteins acting in conjunction with chiral building blocks of the membranes. The microscopic constituents and driving forces that assemble colloidal membranes and lipid membranes are fundamentally different. Despite these differences, both lipid membranes and colloidal membranes are governed by the same elastic energy as both systems have the same set of symmetries. Consequently, our results also point to a phase transition-driven pathway that may be relevant to membrane remodeling.

## Methods

### Virus growth and sample preparation

We purified filamentous virus M13KO7, diameter 6.6 nm, contour length 1.2 μm using standard biological protocol^36^. Purified virus was suspended in 20 mM Tris-HCl (pH = 8.0) buffer containing 100 mM NaCl to screen the electrostatic interactions. Primary amines of the major coat protein of M13KO7 were labeled with amine-reactive fluorophore (DyLight 550 NHS Ester, Thermo Scientific) for fluorescence and confocal microscopy imaging. There are about 3681 labeling sites available on a single virus rod surface. We labeled the virus surface in two different degrees of labeling (2 and 10%, i.e., ~74 and ~368 dye molecules per virus, respectively). Samples were prepared by mixing virus suspension and non-adsorbing polymer polyethylene glycol (PEG 35k, Sigma-Aldrich) and injecting the resultant suspension into a sample chamber. The chamber was prepared using a microscope slide and a coverslip with a thin spacer of parafilm placed between two. Finally, sample chamber was sealed with an epoxy glue to minimize evaporation. Glass slides and coverslips were thoroughly cleaned with hot 1% soap solution (Hellmanex; Hellma Analytics). Coverslips were further treated with ethanol and potassium hydroxide. Cleaned coverslips were coated with a polyacrylamide brush to suppress the depletion interaction between viruses and glass surfaces. The PEG concentration was varied from 20 to 24 mg ml^−1^ for obtaining the equilibrium phase behavior. For all experiments except those involving edge-on membranes, final concentration of virus in the sample was 1.5 mg ml^−1^. Edge-on membranes used to rule out the effect of the substrate were self-assembled at a virus concentration of 5 mg ml^−1^, wherein there is a propensity for few membranes to grow perpendicular to the plane of the coverslip. Crystallization kinetics was followed by preparing samples with PEG concentration at 22 mg ml^−1^ along with temperature variation from 30 to 15 °C. Decrease in temperature increases the PEG osmotic pressure, which leads to solidification of the fluid membrane. A typical sample chamber contains more than 100 membranes with varying sizes, which statistically start crystallizing through nucleation and growth around 20 °C.

### Small angle x-ray scattering measurement

Virus polymer mixtures were injected into a 0.7 mm glass capillaries (Hampton Research), which were then sealed with flame. The capillaries were exposed to X-rays (Hecus S3-Micro system equipped with a 1D position-sensitive detector, HECUS PSD50M) for about an hour and half to obtain well-averaged SAXS intensity vs. scattering vector (*q*) profiles.

### Optical microscopy methods

The differential interference contrast and fluorescence images of membranes were recorded using an inverted microscope (Olympus IX73) equipped with an oil-immersion objective (1.3 NA, 100X UPlanFL N) and connected to a charged-coupled device camera (Cool Snap HQ2, Photometrics). Solid-state source (Sola light engine, Lumencor) and TRITC filter cube (excitation wavelength 511–551 nm, emission wavelength 573–613 nm) were used for fluorescence imaging.

We doped colloidal membranes of unlabeled viruses with fluorescently labeled viruses for the individual rod-tracking measurements. The ratio of number of labeled to unlabeled viruses in these membranes was about 1:30,000. The degree of labeling of individual fluorescent viruses was 10%. Each fluorescently labeled virus appears as an isolated bright spot. The fluorescence images were recorded at 100 ms exposure time.

3D images of solid membranes were obtained by imaging their cross sections using laser scanning confocal microscopy setup (Zeiss LSM 880 Airyscan). System optimized step size value was used for spacing of cross sections. Samples were prepared using fluorescently labeled viruses with 2% degree of labeling. Fluorescence images were acquired by excitation with a 543 nm He-Ne laser. Raw images were stacked and rendered using Matlab.

### Measurement of deformation amplitude

Fluid colloidal membranes are typically conjoined to other membranes through liquid crystalline defects such as pi-walls and pores^37^. We used optical tweezers to slice a larger colloidal membrane into a smaller isolated one or slice through the liquid crystalline defects to obtain an isolated membrane of desired size. Consequently, it is not practically feasible to obtain membranes of identical size repeatedly. The isolated membranes were crystallized by lowering the temperature below the room temperature post-which 3D confocal images were recorded. The deformation amplitude was measured using image processing Matlab codes. The data obtained were then binned to compute a mean deformation corresponding to a mean membrane size. The standard deviation of a particular bin was used as the error bar for that bin.

### Roughness exponent analysis

DIC images of broken solid membrane edges were recorded using the optical microscopy setup described above. Appropriate threshold was used in Matlab image processing programs to detect the jagged profile of the broken edge, *Y*(*X*). The roughness exponent was calculated using root mean square correlation method. We computed standard deviation of *Y*(*X*) for a given window size of length *l*. The window was then scanned across the profile to obtain an average value of standard deviation, $\left\langle {\sigma \left( l \right)} \right\rangle$, for a given *l*. The window size for two given end points (*Y*_2_, *X*_2_) and (*Y*_1_, *X*_1_) was calculated by $l = \sqrt {\left( {Y_2 - Y_1} \right)^2 + \left( {X_2 - X_1} \right)^2}$ to account for the fact the mean profiles were not always along the horizontal/*X*-axis.

### Determination of membrane area covered by peaks and troughs

The topography of the membranes was characterized by height maps, *h*(*x*, *y*) generated from the confocal 3D stacks using ImageJ Plugin Extended Depth of Field. These were further smoothened out using Matlab image processing programs to remove image acquisition noise. The mean surface of membranes was obtained by fitting a polynomial to the height map. Absolute value of the difference between height map value for each pixel and the corresponding value of the fitted mean surface was computed. This matrix was renormalized to have values between 0 and 1 so that the point belonging to the tallest peak had a value equal to 1. All points in the matrix with value larger than 0.35 were said to be belonging to a local peak/trough. Fraction of membrane area covered by local peaks/troughs, *ϕ*, was simply given by total number of points belonging to a local peak/trough divided by total number of points in the height map. The same threshold of 0.35 was used for determination of *ϕ* in all the samples. The numerical values of *ϕ* as shown in Fig. 4e change as this threshold is changed. However, the trend of data remains the same, namely, the area fraction covered by peaks and troughs undergoes a significant jump when number of nucleation centers is increased from 1 to 2.

### Tuning the chirality of colloidal membranes

M13KO7 and M13KO7-Y21M are oppositely handed viruses with identical length. M13KO7-Y21M has three times higher persistence length and significanlty weaker chirality compared to that of M13KO7 (the cholesteric pitch of bulk M13KO7-Y21M samples is four times larger or more compared to that of bulk M13KO7 samples at similar concentrations)^33^. We, therefore, prepared uniform mixtures of M13KO7-Y21M and M13KO7 in the 8:2 stoichiometric ratio to determine the effect of lowering the chirality on the observed curvature instabilities. The two kinds of viruses remained uniformly mixed within the colloidal membranes assembled from the above mentioned mixtures. The membranes were assembled using 18 mg ml^−1^ of PEG and 178 mM NaCl in the suspension buffer. M13KO7-Y21M viruses were fluoresecently labeled with a degree of labeling of 2% to enable confocal microscopy of such membranes.

### Data availability

The data that support the findings of this study are available from the corresponding author on request.

## Electronic supplementary material

Supplementary Information

Description of Additional Supplementary Files

Supplementary Movie 1

Supplementary Movie 2

Supplementary Movie 3

Supplementary Movie 4

Supplementary Movie 5

Supplementary Movie 6

Supplementary Movie 7

Supplementary Movie 8

Supplementary Movie 9

Supplementary Movie 10
