# Supplementary material for: Curvature instability of chiral colloidal membranes on crystallization
Source: Nat Commun. 2017 Oct 27;8:1160. doi: 10.1038/s41467-017-01441-3 (PMC5658384; doi:10.1038/s41467-017-01441-3)
Supplement: Supplementary file 1 — Supplementary Information [file 41467_2017_1441_MOESM1_ESM.pdf]

Supplementary Note 1. Monte-Carlo Simulation

We focus on the multiple grain like structures bounded by ridges/protrusions seen during crystallization of the flat fluid membrane. The ridges persist till the solidification front reach the membrane boundary, post which the membrane undergoes global buckling. The rods in the solid part of the membrane show hexagonal ordering although it is unclear whether the lattice orientation differ in different grains. At least in the case when crystallization starts from one nucleation center, it is likely that the neighboring grains have very small, if not zero, misorientation angle. This is also indirectly supported by the observation that the ridges separating the grains rapidly vanish, annealing the grains, when the solidification front reach the membrane boundary. In case of more than one nucleation center, of course, more lattice orientations are expected with arbitrary misorientation angles. Quite consistently some of the ridges are observed to persist even after global buckling occurs. The grains however are clearly identified by change in the molecular orientation of the virus rods at the walls, in particular their inclination with the membrane normal.

For simplicity, we decouple the orientational degrees of freedom from the translational degree of freedom of the virus rods. We take them to be arranged on a fixed hexagonal lattice and ask, what kind of orientation patterns they exhibit. In fact this simplified model reproduces many of the experimentally observed features as we describe below. For discussion, we rewrite the energy function already discussed in the main text.

$$H = -\epsilon_{\rm N}\sum_{nn}(\hat{\mathbf{n}}_i.\hat{\mathbf{n}}_j)^2 + \epsilon_{\rm C}\sum_{nn}\{[\hat{\mathbf{r}}_{ij}.(\hat{\mathbf{n}}_i\times\hat{\mathbf{n}}_j)](\hat{\mathbf{n}}_i.\hat{\mathbf{n}}_j) - q\}^2 + \epsilon_{\rm D}\sum_{i}(\sin\theta_i)^2. \qquad (1)$$

Here nn stands for sum over all nearest neighbour pairs (i, j). The nematic directors $\hat{\mathbf{n}}_i$, at the $i-th$ site, are represented by their angular orientations (θ_i, ϕ_i). The sign of the depletion term here appears different compared to Eq.1 of main text because we used $(\hat{\mathbf{n}}_i.\hat{\mathbf{z}})^2 = (\cos\theta_i)^2$. We consider total 2314 virus rods arranged on a hexagonal lattice, bounded by a circle. Here $\epsilon_{\rm N}, \epsilon_{\rm D}$ and $\epsilon_{\rm C}$ have dimensions of energy; we work with dimensionless parameters $\epsilon_{\rm C}/\epsilon_{\rm N} = 100, \epsilon_{\rm D}/\epsilon_{\rm N} = 0.6$ and $T \equiv \frac{k_{\rm B}T}{\epsilon_{\rm N}}$. In each Monte-Carlo step our trial rotation angles are $\Delta\theta = \Delta\phi = \pm 1^o$. Whenever θ goes beyond $\pi/2$ we transform $\hat{\mathbf{n}}$ to $-\hat{\mathbf{n}}$ retaining nematic symmetry of the energy function.

The first term of the energy function above is borrowed from the standard Lebwohl-Lasher model [1] for uniaxial nematics with nearest neighbor interaction $U_{ij} = -\epsilon P_2(\theta_{ij})$.

Here θ_{ij} is the angle between the directors at neighboring sites i and j, and $P_2 = \frac{3}{2}\cos^2\theta_{ij} - \frac{1}{2}$ is the 2nd Legendre polynomial. This term accounts for both splay and bend distortions within one Frank constant approximation. Originally this was proposed as a tensorial order parameter in Maier-Saupe theory of phase transitions [2].

We impose torque free boundary condition at the membrane boundary ($r = R_N$) following the Ref. [3, 4]. Our hexagonal lattice ends at the circular boundary. The rods at the boundary ($r = R_N$) are set to lie in the x-y plane (i.e., $\theta = \pi/2$) and are aligned tangentially to the membrane edge (i.e., along $\hat{\boldsymbol{\phi}}$). The last but one layer of rods, at $r = R_{N-1}$, lie in the $(\hat{\mathbf{z}}, \hat{\boldsymbol{\phi}})$ plane maintaining a relative inclination $\delta\theta$ with the rods at the last layer (R_N). Here $R_N - R_{N-1} = \delta r$ is the radial gap between two consecutive concentric layers of rods. The relative angle $\delta\theta$ is determined by the discretized form of the boundary condition $\frac{d\theta}{dr} + \frac{\sin(2\theta)}{2r} = q$. Once the rods at $r = R_N$ and R_{N-1} are fixed now the rest of the system ($R_j, j = 0, N-2$) evolves under our Monte-Carlo simulation. Furthermore, we also switched off the depletion term for the last but two layer (at R_{N-2}) since effect of depletion is known to be weak at the membrane edge.

The emergence of the grain boundaries (ridges)(Supplementary Fig. 2 and Supplementary Fig. 3) is the result of competition among the three terms in the energy function (Supplementary Eq.1). Note that the state with all the directors pointing along $\hat{\mathbf{z}}$ is not a ground state of this model because although, in this state,the 1st and the 3rd terms are minimised (contributing $-3\epsilon_{\mathrm{N}}$), while the 2nd term (preferred chirality) gives a positive contribution $3\epsilon_{\mathrm{C}}q^2$. On the the other hand, the 2nd term is minimised when preferred chiral ordering q is maintained, however it comes with an energy cost due to the 1st and the 3rd terms. When the depletion effect dominates (high ϵ_{D}) over the chiral term, the state with all up (vertical) orientation wins, except at the membrane boundary where effect of the depletion is weak and chirality shows up (Supplementary Fig. 4a,b). The interior of the grains have lower energy than the grain boundaries (Supplementary Fig. 5 b,c). Local energy density of the system shown in Supplementary Fig. 5 b,c indicates dominance of the chiral energy. While the chiral energy term is minimized at grain interiors and at most of the membrane edge, it has high value at the grain boundaries. Supplementary Fig. 5c shows the local energy density map contributed by only the 2nd term of the energy function. As shown in Supplementary Fig. 5d, the interior of the grains have nonzero chirality which minimizes the 2nd term but does not minimise the 1st and the 3rd terms.

The size of the grains is dictated by q (see Supplementary Fig. 2a,b,c or equivalently Supplementary Fig. 3a,b,c): higher the q smaller the domain size. Preferred chirality enforces how slowly, in space, the directors turn (i.e., orientation gradient) from their vertical orientation at the core of the grain to nearly horizontal orientation at the grain boundaries. The grain boundaries in our simulation are π walls. In the experiment, however, the grain boundaries buckle in the third dimension and thereby the rods avoid becoming horizontal. Our model being 2D cannot address this buckling phenomenon.

We note that the grain boundaries in the experiment grow predominantly along the radial direction away from the nucleation site. They bifurcate frequently, making nearly symmetric three way junctions (120° between any two arms). Such junctions are clearly visible in all our simulated structures, see Supplementary Fig. 2, for example.

The sign of the chirality (left or right handed) is not apparent in either experimental plots or our simulation figures, Supplementary Fig. 2 and Supplementary Fig. 3 which show z and $x - y$ projections of the rods, respectively. We, therefore, numerically evaluated the expression for chirality $[\hat{\mathbf{r}}_{ij}.(\hat{\mathbf{n}}_i \times \hat{\mathbf{n}}_j)](\hat{\mathbf{n}}_i.\hat{\mathbf{n}}_j)$ at all lattice points and verified that they all maintain the same sign as imposed by the preferred chirality q.

Several improvements of this model are possible. For example, generalisation to an off-lattice version of this model is possible using Gay-Berne [5] type distance dependent interaction potentials. Such a model we believe will be able to capture the details of the fluid-solid transition observed in the experiment. Incorporating buckling in our model is a difficult problem and will be addressed in the future.

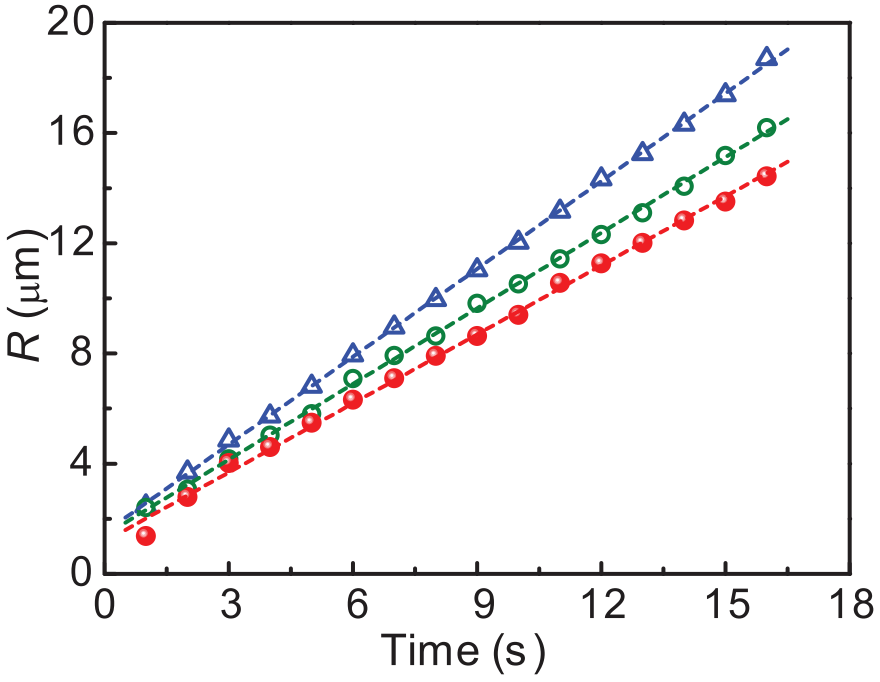

Supplementary Figure 1: Growth kinetics of crystalline domains. Experimental data for the size of the crystalline domain as a function of time at 16^0 C (triangles), 18^0C (open circles), and 19^0C (closed circles).

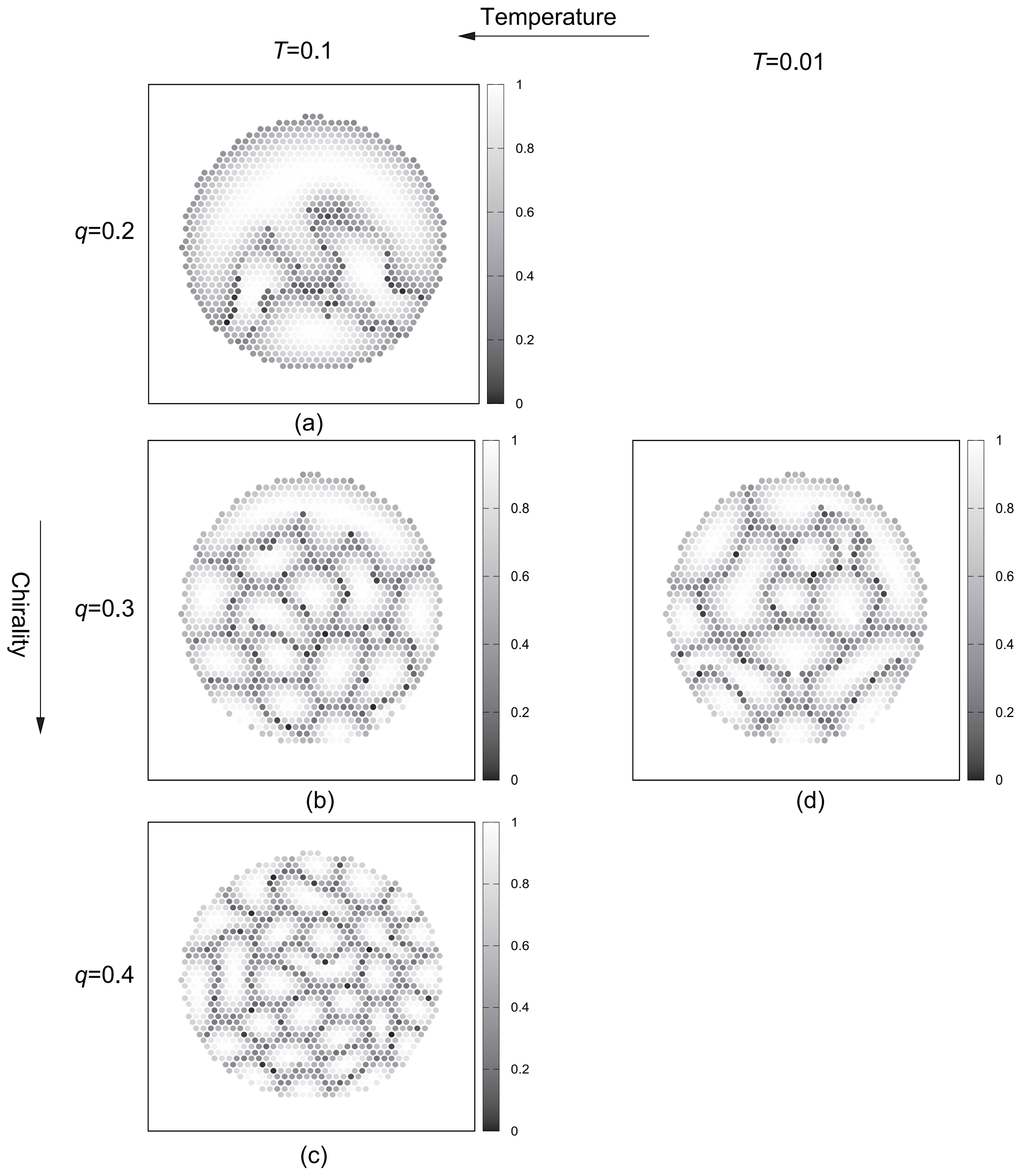

Supplementary Figure 2: Effect of temperature and chirality on the z-component of the directors. MC simulation results showing the variation of the grains and grain boundary structures with preferred chirality q (a, b and c) and temperature (b and d). The thick arrows indicate the direction of increasing temperature or q value, respectively. z-component of the directors' orientation i.e., $(\hat{\mathbf{n}}_i)_z = \cos\theta_i \in [0, 1]$ are plotted. The side bars give the shading scheme. Higher q value favours smaller domain size, and vice versa. However, temperature does not affect the domain sizes.

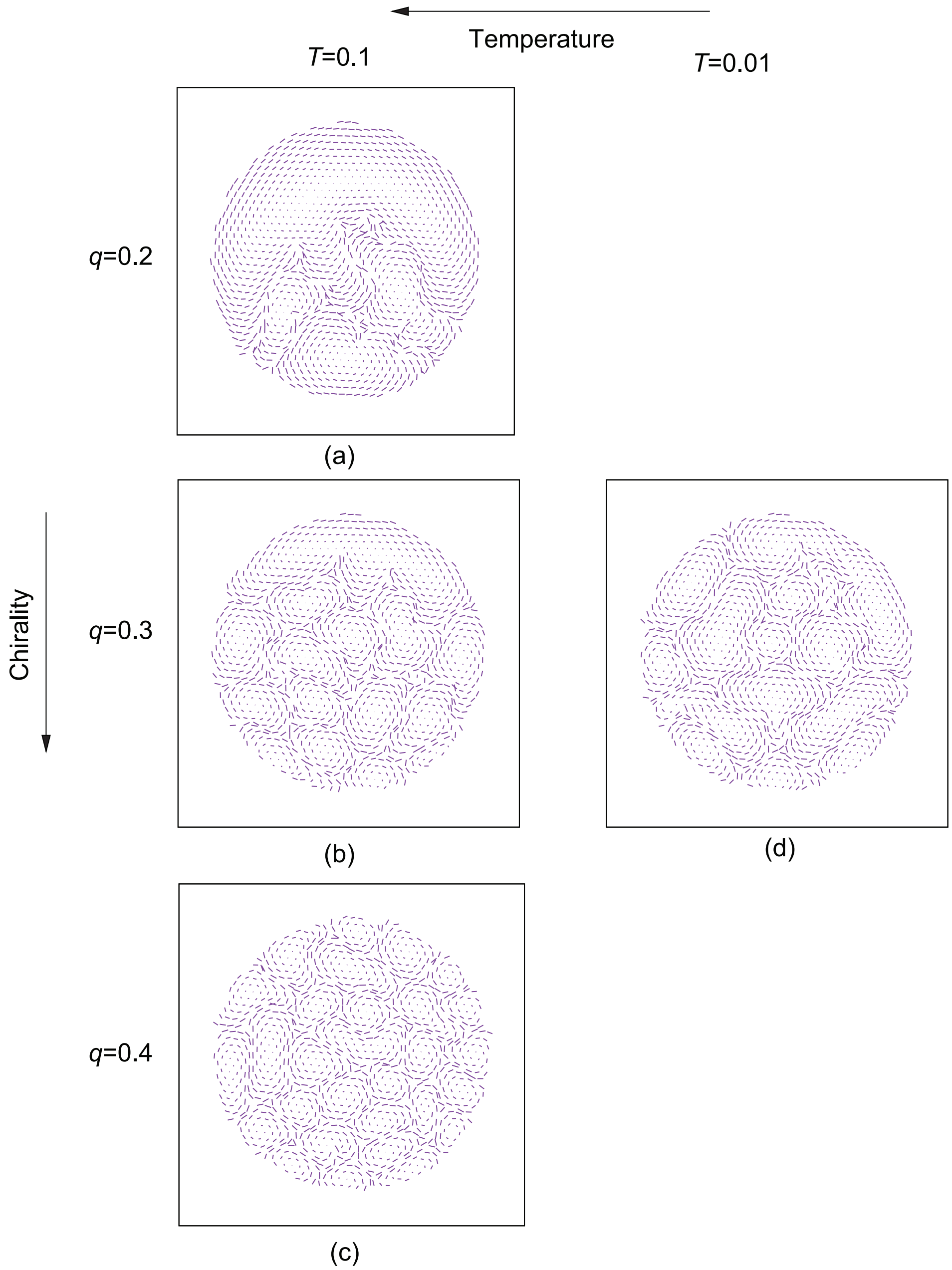

Supplementary Figure 3: Effect of temperature and chirality on the xy-component of the directors. MC simulation results showing the variation of the grains and grain boundary structures with preferred chirality q (a, b and c) and temperature (b, d) and, shown in terms of the projection of the directors in the x-y plane, i.e., perpendicular to the membrane normal. The same director configurations, as in Supplementary Fig. 2, are used.

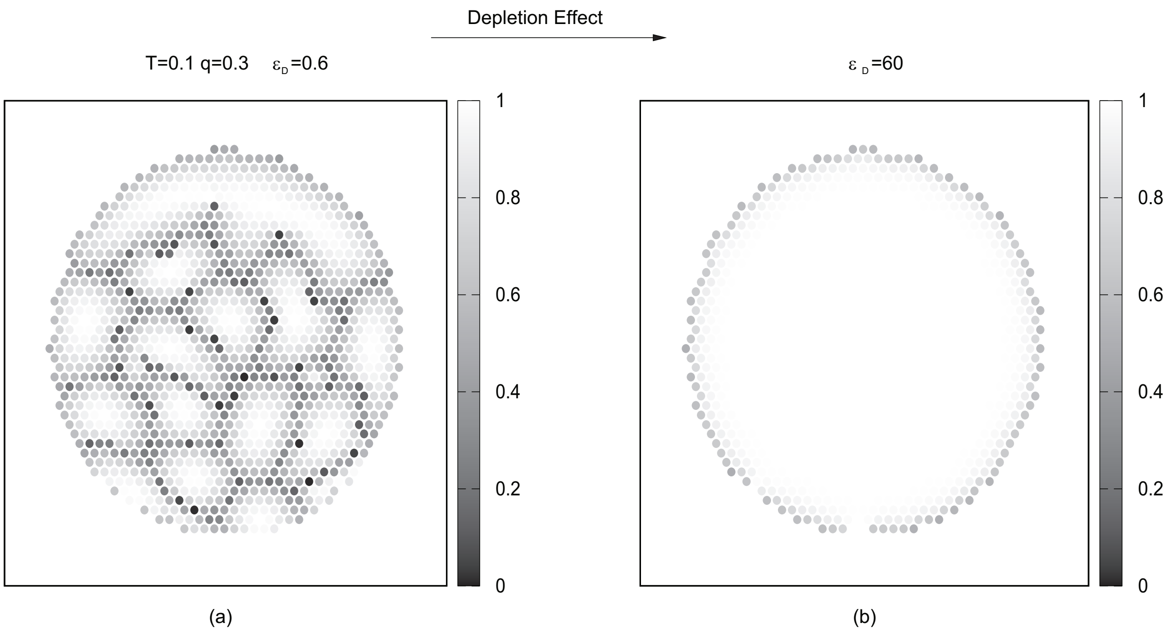

Supplementary Figure 4: Effect of depletion interaction on grain structure. MC simulation results showing change in grain structure due to changes in depletion interaction strength. (a) has a lower value of the depletion coefficient ϵ_D compared to (b). Higher depletion coefficient prevents the formation of grain boundaries.

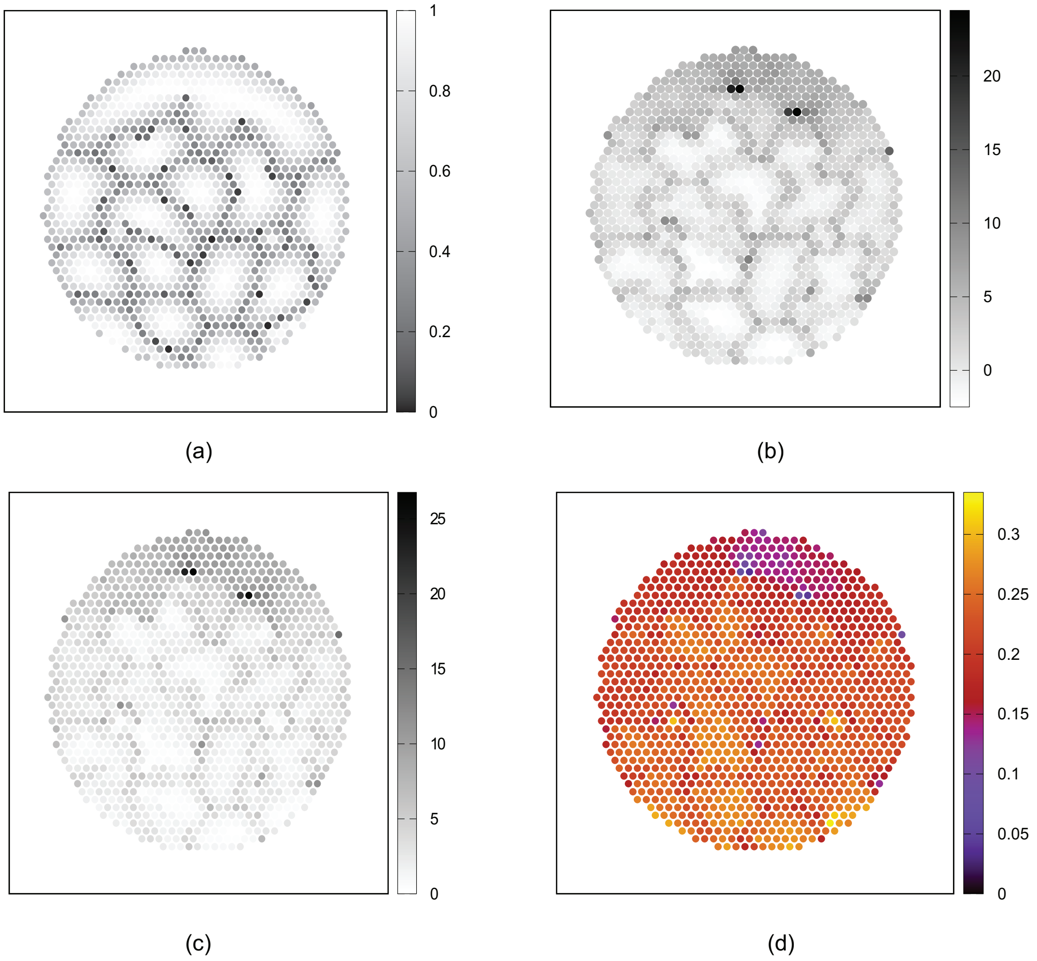

Supplementary Figure 5: Energy density map of crystalline membranes and contribution of chirality in it. MC simulation results for (a) z-component of the directors' orientation $(\hat{\mathbf{n}}_i)_z$ for $q = 0.3$, $T = 0.1$. (b) Local energy density map corresponding to the configuration in (a). (c) Contribution of the chiral term $E_c(i) = \frac{\epsilon_C}{2} \sum_{j \in (nn \text{ of } i)} \{[\hat{\mathbf{r}}_{ij}.(\hat{\mathbf{n}}_i \times \hat{\mathbf{n}}_j)](\hat{\mathbf{n}}_i.\hat{\mathbf{n}}_j) - q\}^2$ to the energy density of the configuration in (a). (d) Values of the local chirality $C(i) = \frac{1}{6} \sum_{j \in (nn \text{ of } i)} [\hat{\mathbf{r}}_{ij}.(\hat{\mathbf{n}}_i \times \hat{\mathbf{n}}_j)](\hat{\mathbf{n}}_i.\hat{\mathbf{n}}_j)$ for the configuration in (a). Most of the domains conform to the preferred chirality q ($= 0.3$ here).

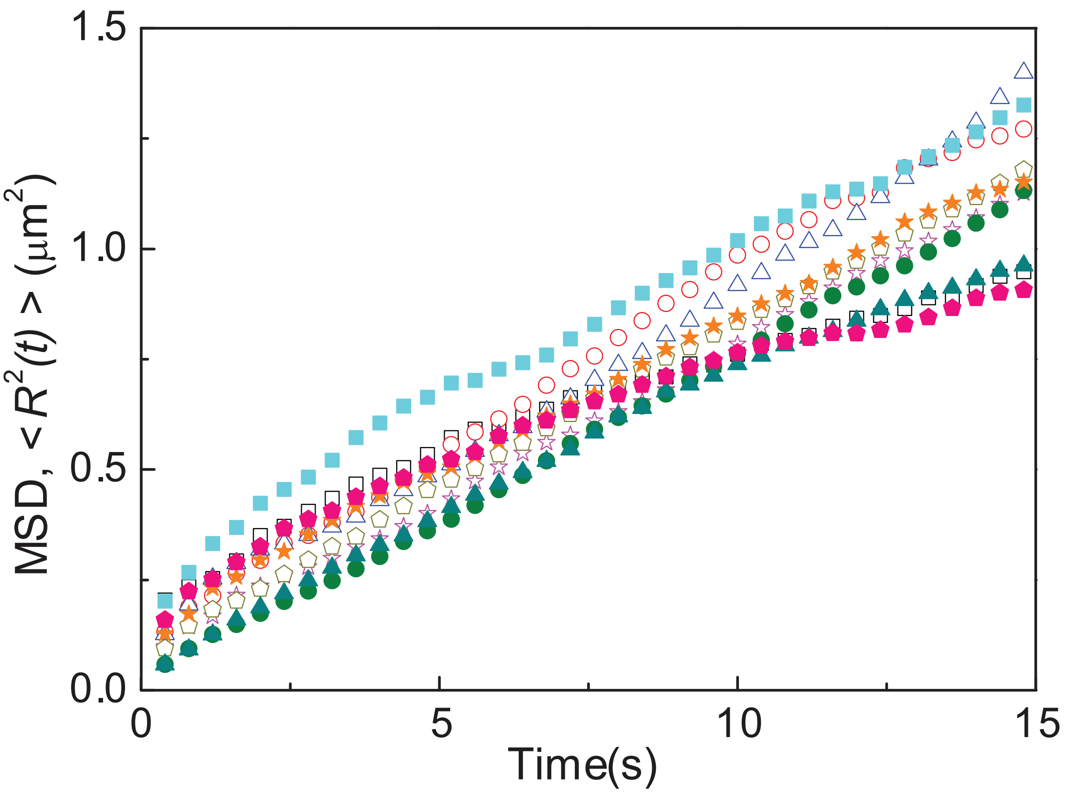

Supplementary Figure 6: Mean Squared Displacement as a function of time for rods at the edge of the crystalline membranes on different days of a given sample. Open squares, circles, triangles, stars, diamonds correspond to days 1 to 5 respectively. Closed squares, circles, triangles, stars, diamonds correspond to days 6 to 10 respectively. Each of the curves corresponding to a given day had been obtained by averaging over data of five rods belonging to different membranes within the sample. Error bars have not been shown to maintain clarity in the display of the data.

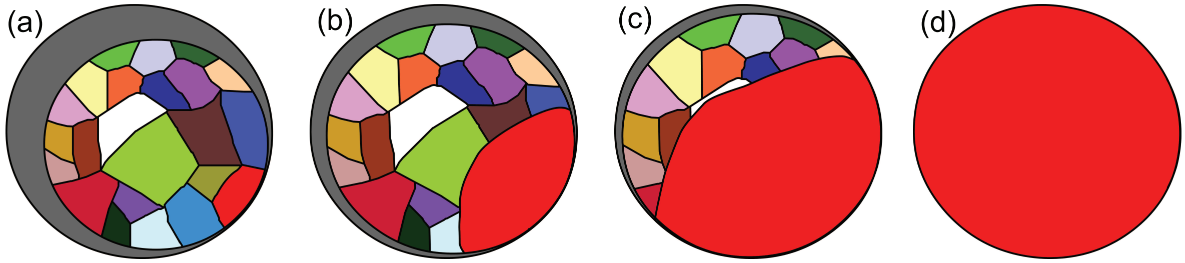

Supplementary Figure 7: Grain boundary migration. (a)-(d) show a schematic of progression of grain boundary migration with increasing time. Each grain is colored in a different color for easy identification. Boundaries between the grains where rods are tilted are denoted with black lines. Grey colored area in (a)-(c) denote the fluid phase.

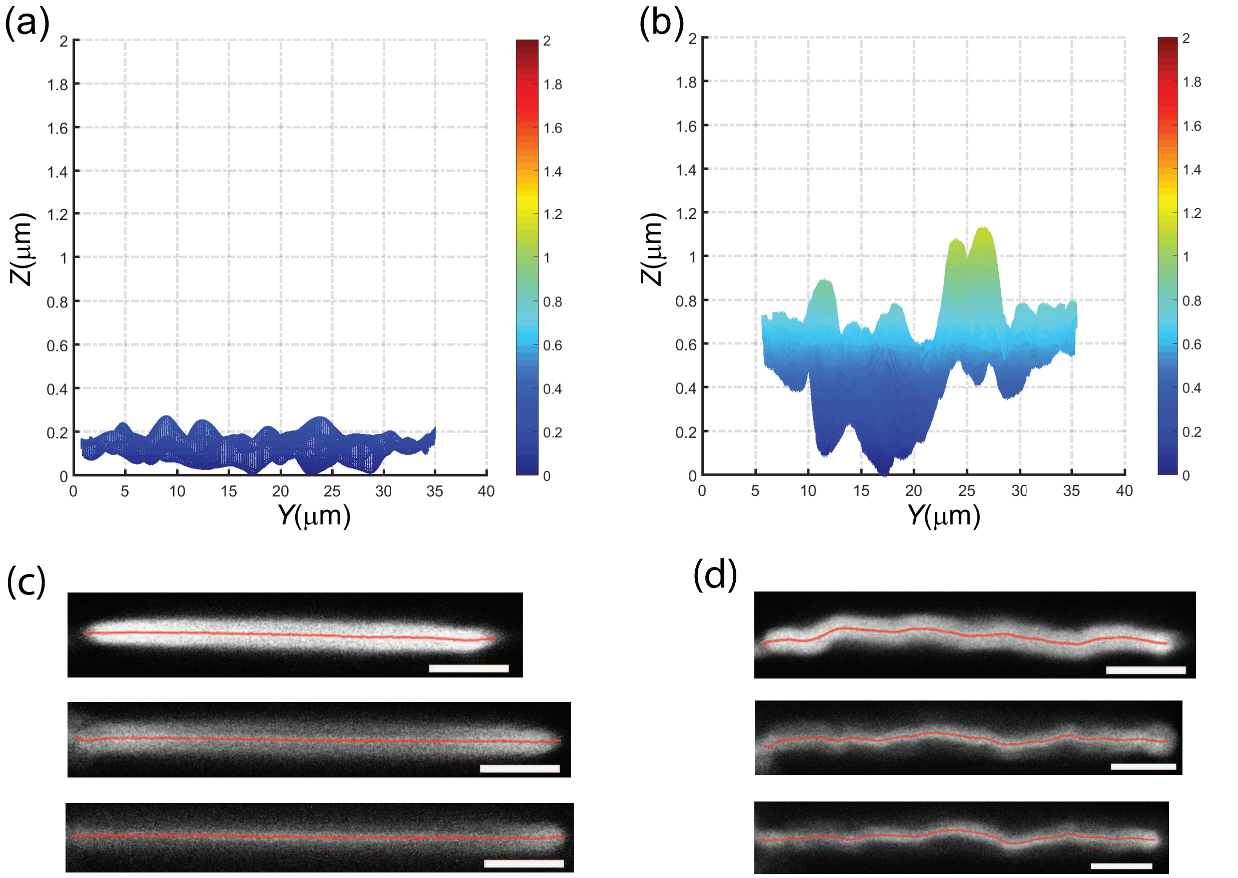

Supplementary Figure 8: Substrate plays negligible role in the curvature instability. 3D confocal rendered images of edge-on membranes in (a) fluid and (b) crystalline phases. The fluid and crystalline membranes were oriented such that their layer normal were in the plane of the cover-slip which was the Y-Z plane. Representative 2D image slices of 3D confocal data of edge-on (c) fluid (d) crystalline membranes. Red lines are the subpixel fits to the position of the membrane plane. Scale bar, 5 μm.

Supplementary References

[1] Lebwohl, P. A. & Lasher, G. Nematic-liquid-crystal ordera Monte Carlo calculation. *Phys. Rev. A* **6**, 426-429 (1972).

[2] Maier, W. & Saupe, A. *Z. Naturforsch A* A simple molecular-statistical theory of the nematic crystalline-liquid phase part-1. **14**, 882-889 (1959).

[3] Pelcovits, R. A & Meyer, R. B. Twist penetration in single-layer smectic A discs of colloidal virus particles. *Liquid Crystals* **36**, 1157-1160 (2009).

[4] Xie, S., Hagan, M. F & Pelcovits, R. A. Interaction of chiral rafts in self-assembled colloidal membranes. *Phys. Rev. E* **93**, 032706 (2016).

[5] Gay, J. G. & Berne, B. J. Modification of the overlap potential to mimic a linear sitesite potential. *J. Chem. Phys.* **74**, 3316-3319 (1981).
