# Supplementary material for: Curvature instability of chiral colloidal membranes on crystallization
Source: Nat Commun. 2017 Oct 27;8:1160. doi: 10.1038/s41467-017-01441-3 (PMC5658384; doi:10.1038/s41467-017-01441-3)
Supplement: Supplementary file 2 — Description of Additional Supplementary Files [file 41467_2017_1441_MOESM2_ESM.pdf]

Description of Additional Supplementary Files

File Name: Supplementary Movie 1
Description: This movie shows the fluid phase of colloidal membranes assembled at 20 mg ml-1 PEG concentration and the solid phase assembled at 25 mg ml-1 PEG concentration. The fluid membrane takes up circular shape and its edge exhibits thermal fluctuations at macroscopic length scales. On the other hand, the solid membrane is highly roughened, buckled and noncircular in shape. There are no observable fluctuations of the edge of the solid membrane. The fluctuations within the bulk of solid membrane, evident to the eye, are tilt and protrusion fluctuations of the constituent rods

File Name: Supplementary Movie 2
Description: This movie shows nucleation and growth of the solid phase within the fluid phase of the colloidal membrane on lowering of the temperature from 30 0C to 15 0C. When the temperature is raised back to 30 0C, the solid phase melts into the fluid phase showing that the transition is reversible.

File Name: Supplementary Movie 3
Description: This movie shows fluorescence time lapse of fluid and solid colloidal membranes (top view) in which 1 out of 30,000 rods are fluorescently labeled. Therefore, individual rods pointing out of the screen appear as bright red spots. The rods freely diffuse within the membrane in the fluid phase. A part of the solid membrane (left side) is out of focus due to out of plane buckling and therefore appears hazy. The rods within the bulk of the solid membrane are immobile. However, the rods at the edge of the membrane continue to diffuse. Individual rods pointing out of the screen appear as bright red spots and those in the plane of the screen appear as lines. The edge bound rods undergo complex dynamics of attachment (bright spot)/ detachment (bright line) to the bulk of the solid phase. These events are highlighted in the movie within encircled area.

File Name: Supplementary Movie 4
Description: This movie shows growth of solid phase from single nucleation center. Post transformation, the membrane is locally flat, but globally buckled. This can be seen from the out of focus part of the membrane near right top corner of the movie.

File Name: Supplementary Movie 5
Description: This movie shows crystallization of a membrane from two nucleation centers located at two opposite halves of the membrane. A large domain wall gets trapped nearly along the diameter of the membrane which buckles out of plane.

File Name: Supplementary Movie 6
Description: This movie shows crystallization of a membrane from three nucleation centers. Many domain walls get trapped within the membrane forming ridges and thus enhance the local surface roughness.

File Name: Supplementary Movie 7
Description: This movie shows crystallization of a membrane from four nucleation centers. Like Supplementary Movie 6, multiple domain walls get trapped within the membrane.

File Name: Supplementary Movie 8
Description: This movie compares the 3-D confocal rendered images of solid colloidal membranes with diameters 8 μm and 46 μm. Each of the membranes was crystallized through a single nucleation center

File Name: Supplementary Movie 9
Description: This movie shows melting of a solid broken membrane having very sharp broken edge into the fluid phase when temperature is increased to 45 0C from room temperature

File Name: Supplementary Movie 10
Description: This movie shows crystallization of a nearly achiral membrane, comprising of left and right-handed chiral rods with a stoichiometric ratio of 2:8, through nucleation and growth. Unlike chiral membranes, these remain flat during and post crystallisation. Crystallization begins with nucleation at 7-8s time-stamp in the movie and is in the well-developed growth phase at 11 s. The entire transformation is completed by 16 s. These events are not immediately obvious to the eye, while viewing the movie, due to complete absence of any out of plane deformation.
